# Exploration of phylogeography of *Monachacantiana* s.l. continues: the populations of the Apuan Alps (NW Tuscany, Italy) (Eupulmonata, Stylommatophora, Hygromiidae)

**DOI:** 10.3897/zookeys.814.31583

**Published:** 2019-01-09

**Authors:** Joanna R. Pieńkowska, Giuseppe Manganelli, Folco Giusti, Debora Barbato, Alessandro Hallgass, Andrzej Lesicki

**Affiliations:** 1 Department of Cell Biology, Institute of Experimental Biology, Faculty of Biology, Adam Mickiewicz University in Poznan; Umultowska 89, 61-614 Poznań, Poland Adam Mickiewicz University in Poznan Poznań Poland; 2 Dipartimento di Scienze Fisiche, della Terra e dell’Ambiente, Universitá di Siena, Via Mattioli 4, 53100 Siena, Italy Universitá di Siena Siena Italy

**Keywords:** 16SrDNA, COI, H3, ITS2, molecular features, shell and genital structure, species distribution

## Abstract

Two new lineages CAN-5 and CAN-6 were recognised in four populations of *Monachacantiana* (Montagu, 1803) s.l. from the Italian Apuan Alps by joint molecular and morphological analysis. They are different from other *M.cantiana* lineages known from English, Italian, Austrian and French populations, i.e. CAN-1, CAN-2, CAN-3 and CAN-4, as well as from the other Italian *Monacha* species used for comparisons (*M.parumcincta* and *M.cartusiana*). Although a definite taxonomic and nomenclatural setting seems to be premature, we suggest that the name or names for these new lineages as one or two species should be found among 19^th^ century names (*Helixsobara* Mabille, 1881, *H.ardesa* Mabille, 1881, *H.apuanica* Mabille, 1881, *H.carfaniensis* De Stefani, 1883 and *H.spallanzanii* De Stefani, 1884).

## Introduction

Study of the phylogeography of the *Monachacantiana* (Montagu, 1803) s.l. by joint molecular and morphological analysis revealed a number of cryptic lineages, some of which might deserve distinct taxonomic status.

Examination of a first group of English, Italian, Austrian and French populations showed that it consisted of at least four distinct lineages (CAN-1, CAN-2, CAN-3, CAN-4) (Pieńkowska et al. 2018). One of these lineages (CAN-1) included most of the UK (5 sites) and Italian (5 sites) populations examined. Three other lineages were represented by populations from two sites in northern Italy (CAN-2), three sites in northern Italy and Austria (CAN-3) and two sites in south-eastern France (CAN-4). A taxonomic and nomenclatural setting is only currently available for CAN-1 and CAN-4. The lineage CAN-1 corresponds to the true *M.cantiana* (Montagu, 1803) because it is the only one that includes topotypical English populations. The lineage CAN-4 is attributed to *M.cemenelea* (Risso, 1826), for which a neotype has been designated and deposited. A definitive frame for the other two has been postponed because it requires much more research.

We have now studied some populations from the Apuan Alps at the north-western extremity of Tuscany, a well-known hotspot of diversity and endemism (Lanza 1997; Biondi et al. 2013; Garbari and Bedini 2014; Carta et al. 2017; Orsenigo et al. 2018). Molecular study revealed two more lineages (CAN-5 and CAN-6), molecularly distinct from each other and from all the others, but morphologically indistinguishable from each other and only slightly distinguishable from all the other lineages of *M.cantiana*.

## Material and methods

### Taxonomic sample

Four new populations of *Monachacantiana* s.l. were considered in our analysis of their molecular and morphological (shell and genitalia structure) variability (Table 1) and compared with the other *M.cantiana* lineages (Pieńkowska et al. 2018). The sequences deposited in GenBank were also considered for the molecular analysis. Two other *Monacha* species were used for molecular comparison (*Monachacartusiana* (Müller, 1774)) and for morphological and molecular comparison (*M.parumcincta* (Rossmässler, 1834)).

**Table 1.** List of localities of the populations of *Monachacantiana* s.l. (CAN-5 & CAN-6) used for molecular and morphological (SH shell, AN genitalia) research.

Localities				Clade	Revised taxonomy	COI		16SrDNA		H3		ITS2		PCA and RDA	Figs
No.	Coordinates	Country and site	Collector / date / no. of specimens (collection)			New haplotype (no. spec.)	GenBank ##	New haplotype (no. spec.)	GenBank ##	New common sequence (no. sps)	GenBank ##	New common sequence (no. sps)	GenBank ##		
1	44°06'54.9"N 10°08'23.9"E	Italy, Tuscany, Apuan Alps, Foce di Pianza (pathway from Campo Cecina to Monte Sagro), 1270 m a.s.l.	A. Hallgass / 13.10.2013 / 5 / (FGC 41565)	CAN-5	*M.* sp.	COI 1 (4)	MK066929	16S 1 (4)	MK066947	H3 5 (3)	MK066965			SH, AN	8, 9, 25–29
							MK066930		MK066948		MK066966	ITS2 1 (1)	MK066981		
							MK066931		MK066949		MK066967	ITS2 6 (1)	MK066982		
							MK066932		MK066950	H3 6 (1)	MK066968	ITS2 2 (1)	MK066983		
						COI 3 (1)	MK066933	16S 5 (1)	MK066951	H3 8 (1)	MK066969	ITS2 5 (1)	MK066984		
2	44°07'21.2"N 10°07'17.7"E	Italy, Tuscany, Apuan Alps, Campo Cecina, 500 m N of Rifugio CAI Carrara, 1300 m a.s.l.	A. Hallgass / 13.10.2013 / 5 / (FGC 41564)	CAN-5	*M.* sp.	COI 4 (3)	MK066934	16S 6 (2)	MK066952	H3 6 (1)	MK066970	ITS2 10 (2)	MK066985	SH, AN	10–12, 30–34
									MK066953	H3 1 (1)	MK066971		MK066986		
							MK066935	16S 7 (1)	MK066954	H3 3 (1)	MK066972	ITS2 5 (2)	MK066987		
							MK066936	16S 8 (1)	MK066955	H3 7 (1)	MK066973		MK066988		
						COI 5 (1)	MK066937	16S 6 (1)	MK066956	H3 2 (1)	MK066974	ITS2 3 (1)	MK066989		
3	44°05'56.8"N 10°07'08.5"E	Italy, Tuscany, Apuan Alps, Piastra, 290 m a.s.l.	A. Hallgass / 13.10.2013 / 5 / (FGC 41563)	CAN-5	*M.* sp.					H3 6 (2)	MK066975	ITS2 11 (1)	MK066990	SH, AN	6, 7, 19–24
						COI 1 (3)	MK066938	16S 2 (1)	MK066957		MK066976	ITS2 4 (1)	MK066991		
							MK066939	16S 3 (1)	MK066958	H3 5 (1)	MK066977	ITS2 12 (1)	MK066992		
							MK066940					ITS2 2 (1)	MK066993		
						COI 2 (1)	MK066941	16S 4 (1)	MK066959			ITS2 3 (1)	MK066994		
4	44°03'25.5"N 10°16'01.0"E	Italy, Tuscany, Apuan Alps, 1 km E of Campagrina, 769 m a.s.l.	A. Hallgass / 22.10.2011 / 5 (FGC 40322)	CAN-6	*M.* sp.	COI 6 (1)	MK066942	16S 9 (1)	MK066960			ITS2 9 (2)	MK066995	SH, AN	13–15, 35–41
						COI 7 (1)	MK066943	16S 11 (1)	MK066961				MK066996		
						COI 8 (3)	MK066944	16S 10 (1)	MK066962	H3 4 (1)	MK066978	ITS2 8 (1)	MK066997		
							MK066945	16S 12 (2)	MK066963	H3 1 (1)	MK066979	ITS2 13 (1)	MK066998		
							MK066946		MK066964	H3 5 (1)	MK066980	ITS2 7 (1)	MK066999		

### Materials examined

New materials examined are listed as follows, when possible: geographic coordinates of locality, locality (country, region, site, municipality and province), collector(s), date, number of specimens with name of collection where materials are kept in parenthesis (Table 1). The materials are kept in the F. Giusti collection (FGC; Dipartimento di Scienze Fisiche, della Terra e dell’Ambiente, Università di Siena, Italy). The materials used for comparison have already been described (see Pieńkowska et al. 2018: table 1) and is now supplemented with some new nucleotide sequences (Table 2).

**Table 2.** New ITS2 sequences obtained from the specimens of *Monachacantiana* s.l. (CAN-2 to CAN-4) and *M.parumcincta* (PAR) used for molecular research. Number of localities after Pieńkowska et al. (2018). Earlier data on other sequences (COI, 16SrDNA, H3 and ITS2) from these localities were published by Pieńkowska et al. (2018).

Localities				Clade	Revised taxonomy	ITS2		
No.	Coordinates	Country and site	Collector / date / no. of specimens (collection)			New common sequence	No. Spec.	GenBank ##
12	45°11'59.85"N 10°58'49.30"E	Italy, Venetum, Sorgà (Verona)	A. Hallgass / 09.2012 / 6 (FGC 42964)	CAN-2	* M. cantiana *	ITS2 14	1	MK067000
15	44°22'09.98"N 11°15'11.28"E	Italy, Emilia Romagna, along Fiume Setta, upstream its confluence with Fiume Reno (Sasso Marconi, Bologna)	A. Hallgass / 09.2012 / 3 (FGC 42977)	CAN-3	*M.* sp.	ITS2 15	1	MK067001
17	48°15'25.50"N 16°30'46.38"E	Austria, Breitenlee, abandoned railway station	M. Duda / 09.2015 / 3 (FGC 44020)	CAN-3	*M.* sp.	ITS2 16	1	MK067002
18	43°46'11.79"N 07°22'21.50"E	France, Alpes-Maritimes, Vallée de Peillon, Sainte Thecle	A. Hallgass / 24.10.2011/ 5 (FGC 40320)	CAN-4	* M. cemenelea *	ITS2 17	1	MK067003
						ITS2 18	1	MK067004
24	40°13'25.49"N 15°52'17.07"E	Italy, Basilicata, along the road from Moliterno to Fontana d’Eboli (Moliterno, Potenza)	A. Hallgass / 2012 / 5 (FGC 42962)	PAR	* M. parumcincta *	ITS2 19	1	MK067005

### DNA extraction, amplification and sequencing

DNA extraction, amplification and sequencing methods are described in detail in our previous paper (Pieńkowska et al. 2018).

### Phylogenetic inference

Two mitochondrial and two nuclear gene fragments were analysed, namely cytochrome c oxidase subunit 1 (COI), 16S ribosomal DNA (16SrDNA), histone 3 (H3) and an internal transcribed spacer of rDNA (ITS2), respectively. All new sequences were deposited in GenBank (Tables 1, 2). The COI, 16SrDNA, H3 and ITS2 sequences obtained from GenBank for comparisons are listed in Table 3.

**Table 3.** GenBank sequences used for comparison in molecular analysis.

Species	COI	16SrDNA	H3	ITS2	References
*Monachacantiana* CAN-1		KJ458539			Razkin et al. 2015
	KM247375	KM247390			Pieńkowska et al. 2015
	KX507234	KX495428			Neiber and Hausdorf 2015
	MG208884-MG208924	MG208960-MG208995	MG209031-MG209039	MH137963-MH137978	Pieńkowska et al. 2018
			MG209041-MG209048		
*Monachacantiana* CAN-2	MG208925-MG208932	MG208996-MG209004	MG209049-MG209052	MH137979-MH137981	Pieńkowska et al. 2018
*Monachacantiana* CAN-3	HQ204502	HQ204543			Duda et al. 2011; Kruckenhauser et al. 2014
	KF596907	KF596863			Cadahia et al. 2014
	MG208933-MG208938	MG209005-MG209010	MG209040	MH137982-MH137983	Pieńkowska et al. 2018
			MG209053-MG209057		
*Monachacemenelea* CAN-4	MG208939-MG208943	MG209011-MG209015	MG209058-MG209060	MH137984	Pieńkowska et al. 2018
*Monacha* sp.		AY741419			Manganelli et al. 2005
*Monachaparumcincta* PAR		AY741418			Manganelli et al. 2005
	MG208944-MG208959	MG209016-MG209030	MG209061-MG209071	MH137985-MH137992	Pieńkowska et al. 2018
* Monacha cartusiana *	KM247380	KM247397			Pieńkowska et al. 2015
	KX507189	KX495378			Neiber and Hausdorf 2015
			MG209072	MH137993	Pieńkowska et al. 2018

The sequences were edited by eye using the programme BioEdit, version 7.2.6 (Hall 1999). Alignments were performed using Clustal W (Thompson et al. 1994) implemented in MEGA 7 (Kumar et al. 2016). The COI and H3 sequences were aligned according to the translated amino acid sequences. The ends of all sequences were trimmed. The lengths of the sequences after trimming were 591 bp for COI, 355 positions for 16SrDNA, 315 bp for H3 and 496 positions for ITS2. The sequences were collapsed to haplotypes (COI and 16SrDNA) and to common sequences (H3 and ITS2) using the programme ALTER (Alignment Transformation EnviRonment) (Glez-Peña et al. 2010). Gaps and ambiguous positions were removed from alignments prior to phylogenetic analysis. Mitochondrial (COI and 16SrDNA) and nuclear (H3 and ITS2) sequences were combined (Table 4) before phylogenetic analysis. Finally, the sequences of COI, 16SrDNA, H3 and ITS2 were combined (Table 4) for Maximum Likelihood (ML) and Bayesian inference (BI). Before doing so, uncertain regions were removed from 16SrDNA alignment with the GBlocKs 0.91b (Castresana 2000; Talavera and Castresana 2007) using parameters for relaxed selection of blocks. This procedure shortened 16SrDNA sequences from 355 to 275 positions.

**Table 4.** Combined Sequences of the following gene sequences: COI+16SrDNA and H3+ITS2 for ML analysis and of COI+16SrDNA+H3+ITS2 for Bayesian analysis.

Combined sequence	COI haplotype	16S haplotype	Combined sequence	H3 sequence	ITS2 sequence	Combined sequence	COI haplotype	16S haplotype	H3 sequence	ITS2 sequence	Locality
IT-COI16S-1	MK066929	MK066947									Italy, Tuscany, Foce di Pianza
			IT-H3ITS2-6	MK066966	MK066981	IT-CS-1	MK066930	MK066948	MK066966	MK066981	Italy, Tuscany, Foce di Pianza
			IT-H3ITS2-7	MK066967	MK066982	IT-CS-2	MK066931	MK066949	MK066967	MK066982	Italy, Tuscany, Foce di Pianza
			IT-H3ITS2-8	MK066968	MK066983	IT-CS-3	MK066932	MK066950	MK066968	MK066983	Italy, Tuscany, Foce di Pianza
IT-COI16S-2	MK066938	MK066957	IT-H3ITS2-13	MK066976	MK066991	IT-CS-4	MK066938	MK066957	MK066976	MK066991	Italy, Piastra
IT-COI16S-3	MK066939	MK066958				IT-CS-5	MK066939	MK066958	MK066977	MK066992	Italy, Piastra
IT-COI16S-4	MK066941	MK066959									Italy, Piastra
IT-COI16S-5	MK066933	MK066951				IT-CS-6	MK066933	MK066951	MK066969	MK066984	Italy, Tuscany, Foce di Pianza
IT-COI16S-6	MK066934	MK066952	IT-H3ITS2-9	MK066970	MK066985	IT-CS-7	MK066934	MK066952	MK066970	MK066985	Italy, Tuscany, Campo Cecina
IT-COI16S-7	MK066935	MK066954	IT-H3ITS2-11	MK066972	MK066987	IT-CS-8	MK066935	MK066954	MK066972	MK066987	Italy, Tuscany, Campo Cecina
IT-COI16S-8	MK066936	MK066955	IT-H3ITS2-12	MK066973	MK066988	IT-CS-9	MK066936	MK066955	MK066973	MK066988	Italy, Tuscany, Campo Cecina
IT-COI16S-9	MK066937	MK066956	IT-H3ITS2-10	MK066974	MK066989	IT-CS-10	MK066937	MK066956	MK066974	MK066989	Italy, Tuscany, Campo Cecina
IT-COI16S-10	MK066942	MK066960									Italy, Tuscany, Campagrina
IT-COI16S-11	MK066943	MK066961									Italy, Tuscany, Campagrina
IT-COI16S-12	MK066944	MK066962	IT-H3ITS2-4	MK066978	MK066997	IT-CS-11	MK066944	MK066962	MK066978	MK066997	Italy, Tuscany, Campagrina
IT-COI16S-13	MK066945	MK066963									Italy, Tuscany, Campagrina
			IT-H3ITS2-5	MK066980	MK066999	IT-CS-12	MK066946	MK066964	MK066980	MK066999	Italy, Tuscany, Campagrina
UK-COI16S-1	MG208884	MG208966	UK-H3ITS2-1	MG209031	MH137963	UK-CS-1	MG208884	MG208966	MG209031	MH137963	UK, Barrow near Barnsley
UK-COI16S-2	MG208893	MG208960									UK, Rotherham
UK-COI16S-3	MG208899	MG208976	UK-H3ITS2-2	MG209038	MH137971	UK-CS-2	MG208899	MG208976	MG209038	MH137971	UK, Sheffield
UK-COI16S-4	MG208898	MG208975	UK-H3ITS2-3	MG209037	MH137969	UK-CS-3	MG208898	MG208975	MG209037	MH137969	UK, Rotherham
UK-COI16S-5	MG208891	MG208972									UK, Cambridge
IT-COI16S-14	MG208915	MG208985	IT-H3ITS2-15	MG209045	MH137973	IT-CS-13	MG208915	MG208985	MG209045	MH137973	Italy, Latium, Valle dell’Aniene, Rome
IT-COI16S-15	MG208916	MG208987	IT-H3ITS2-16	MG209046	MH137974	IT-CS-14	MG208916	MG208987	MG209046	MH137974	Italy, Latium, Valle dell’Aniene, Rome
IT-COI16S-16	MG208917	MG208989	IT-H3ITS2-17	MG209047	MH137975	IT-CS-15	MG208917	MG208989	MG209047	MH137975	Italy, Latium, Valle dell’Aniene, Rome
IT-COI16S-17	MG208905	MG208977	IT-H3ITS2-18	MG209039	MH137972	IT-CS-16	MG208905	MG208977	MG209039	MH137972	Italy, Latium, Gole del Velino
IT-COI16S-18	MG208906	MG208979									Italy, Latium, Gole del Velino
IT-COI16S-19	MG208921	MG208990				IT-CS-17	MG208921	MG208990	MG209043	MH137976	Italy, Latium, Valle del Tronto
IT-COI16S-20	MG208923	MG208994	IT-H3ITS2-19	MG209048	MH137978	IT-CS-18	MG208923	MG208994	MG209048	MH137978	Italy, Latium, Valle del Turano
IT-COI16S-21	MG208910	MG208978									Italy, Latium, Gole del Velino
IT-COI16S-22	MG208925	MG208996	IT-H3ITS2-22	MG209050	MK067000	IT-CS-19	MG208925	MG208996	MG209050	MK067000	Italy, Venetum, Sorga
IT-COI16S-23	MG208926	MG209001	IT-H3ITS2-21	MG209049	MH137979						Italy, Venetum, Sorga
IT-COI16S-24	MG208928	MG208998									Italy, Venetum, Sorga
IT-COI16S-25	MG208932	MG209003	IT-H3ITS2-20	MG209052	MH137981	IT-CS-20	MG208932	MG209003	MG209052	MH137981	Italy, Lombardy, Rezzato
IT-COI16S-26	MG208934	MG209005	IT-H3ITS2-2	MG209040	MK067001	IT-CS-21	MG208934	MG209005	MG209040	MK067001	Italy, Emila Romagna, Fiume Setta
IT-COI16S-27	MG208933	MG209007	IT-H3ITS2-3	MG209054	MH137982	IT-CS-22	MG208933	MG209007	MG209054	MH137982	Italy, Emila Romagna, Fiume Setta
IT-COI16S-28	MG208944	MG209017	IT-H3ITS2-24	MG209061	MK067005	IT-CS-23	MG208944	MG209017	MG209061	MK067005	Italy, Basilicata, Moliterno to Fontana d’Eboli
IT-COI16S-29	MG208946	MG209019	IT-H3ITS2-23	MG209064	MH137992						Italy, Basilicata, Moliterno to Fontana d’Eboli
IT-COI16S-30	MG208949	MG209020				IT-CS-24	MG208949	MG209020	MG209067	MH137987	Italy, Tuscany, Nievole
IT-COI16S-31	MG208950	MG209028	IT-H3ITS2-25	MG209068	MH137989						Italy, Tuscany, Arezzo
			IT-H3ITS2-26	MG209070	MH137990						Italy, Tuscany, Arezzo
			IT-H3ITS2-27	MG209062	MH137986						Italy, Tuscany, Podere Castella
AT-COI16S-1	MG208936	MG209009	AT-H3ITS2-1	MG209055	MH137983	AT-CS-1	MG208936	MG209009	MG209055	MH137983	Austria, Breitenlee
AT-COI16S-2	MG208938	MG209008									Austria, Breitenlee
FR-COI16S-1	MG208939	MG209011	FR-H3ITS2-1	MG209058	MH137984	FR-CS-1	MG208939	MG209011	MG209058	MH137984	France, Sainte Thecle
FR-COI16S-2	MG208940	MG209012	FR-H3ITS2-2	MG209059	MK067003	FR-CS-2	MG208940	MG209012	MG209059	MK067003	France, Sainte Thecle
FR-COI16S-3	MG208941	MG209013	FR-H3ITS2-3	MG209060	MK067004	FR-CS-3	MG208941	MG209013	MG209060	MK067004	France, Sainte Thecle
HU-COI16S-1	KM247376	KM247391	HU-H3ITS2-1	MG209072	MH137993	HU-CS-1	KM247376	KM247391	MG209072	MH137993	Hungary, Kis-Balaton

The sequences of COI obtained in this study together with other sequences from GenBank were analysed by the genetic distance Neighbour-Joining method (Saitou and Nei 1987) implemented in MEGA7 using the Kimura two-parameter model (K2P) for pairwise distance calculations (Kimura 1980). Maximum Likelihood (ML) analyses were then performed with MEGA 7. *Monachacartusiana* and *Monachaparumcincta* were added as outgroup species in each analysis. For ML analysis of combined sequences, the following best nucleotide substitution models were specified according to the Bayesian Information Criterion (BIC): HKY+G (Hasegawa et al. 1985; Kumar et al. 2016) for COI and 16SrDNA combined sequences of 879 positions (591 COI + 288 16SrDNA), TN92+G (Tamura 1992; Kumar et al. 2016) for H3+ITS2 combined sequences of 812 positions (315 H3 + 497 ITS2), and GTR+I+G (Nei and Kumar 2000; Kumar et al. 2016) for COI+16SrDNA+H3+ITS2 combined sequences with a total length of 1677 positions (591 COI + 275 16SrDNA + 315 H3 + 496 ITS2). Bayesian analysis was conducted with the MrBayes 3.1.2 (Ronquist and Huelsenbeck 2003) using the same evolution model as for ML calculation. The GTR substitution model (Nei and Kumar 2000; Kumar et al. 2016), assuming a gamma distributed rate variation (+G) allowing for some sites to be evolutionarily invariable (+I), was identified as the best-fit substitution model using jModelTest2 (Darriba et al. 2012). Four Monte Carlo Markov chains were run for one million generations, sampling every 100 generations (the first 250,000 trees were discarded as ‘burn-in’). This gave us a 50% majority rule consensus tree. In parallel, Maximum Likelihood (ML) analysis was performed with MEGA7 (Kumar et al. 2016) and calculated bootstrap values were mapped on the 50% majority rule consensus Bayesian tree.

The haplotype network was inferred with Network 5.0.0.1 to reflect all relationships between COI and 16SrDNA haplotypes. During the analysis, a median-joining calculation implemented in Network 5.0.0.1 was used (Bandelt et al. 1999).

### Morphological study

Seventy-eight specimens of seven clades (six lineages of *M.cantiana* s.l.: CAN-1, CAN-2, CAN-3, CAN-4, CAN-5 and CAN-6; one lineage of *M.parumcincta*) were considered for shell variability (see Table 1 and Pieńkowska et al. 2018). Shell variability was analysed randomly choosing five adult specimens from each population, when possible. Twelve shell variables were measured to the nearest 0.1 mm using Adobe Photoshop 7.0.1 on digital images of apertural and umbilical standard views taken with a Canon EF 100 mm 1:2.8 L IS USM macro lens mounted on a Canon F6 camera: AH aperture height, AW aperture width, LWfW last whorl final width, LWmW last whorl medial width, LWaH height of adapical sector of last whorl, LWmH height of medial sector of last whorl, PWH penultimate whorl height, PWfW penultimate whorl final width, PWmW penultimate whorl medial width, SD shell diameter, SH shell height, UD umbilicus diameter (see Pieńkowska et al. 2018: fig. 1).

Seventy-five specimens of seven clades (all lineages of *M.cantiana* s.l. plus one lineage of *M.parumcincta*) were analysed for anatomical variability (see Table 1 and Pieńkowska et al. 2018). Snail bodies were dissected under the light microscope (Wild M5A or Zeiss SteREO Lumar V12). Anatomical details were drawn using a Wild camera lucida. Acronyms: BC bursa copulatrix, BW body wall, DBC duct of bursa copulatrix, DG digitiform glands, E epiphallus (from base of flagellum to beginning of penial sheath), F flagellum, FO free oviduct, GA genital atrium, OSD ovispermiduct, P penis, V vagina, VA vaginal appendix (also known as appendicula), VAS vaginal appendix basal sac, VD vas deferens. Six anatomical variables (DBC, E, F, P, V, VA) were measured using a calliper under a light microscope (0.01 mm) (see Pieńkowska et al. 2018: fig. 2).

Detailed methods of multivariate ordination by Principal Component Analysis (PCA) and Redundancy Analysis (RDA), performed on the original shell and genitalia matrices as well as on the Z-matrices (shape-related matrices), are described in a previous paper (Pieńkowska et al. 2018).

Differences between species for each shell and genital character were assessed through box-plots and descriptive statistics. Significance of differences (set at *p* ≤ 0.01) was obtained using analysis of variance (ANOVA); when the test proved significant, an adjusted posteriori pair-wise comparison between pairs of species was performed using Tukey’s honestly significant difference (HSD) test. All variables were log transformed before analysis.

## Results

### Molecular study

**Figure 1.**
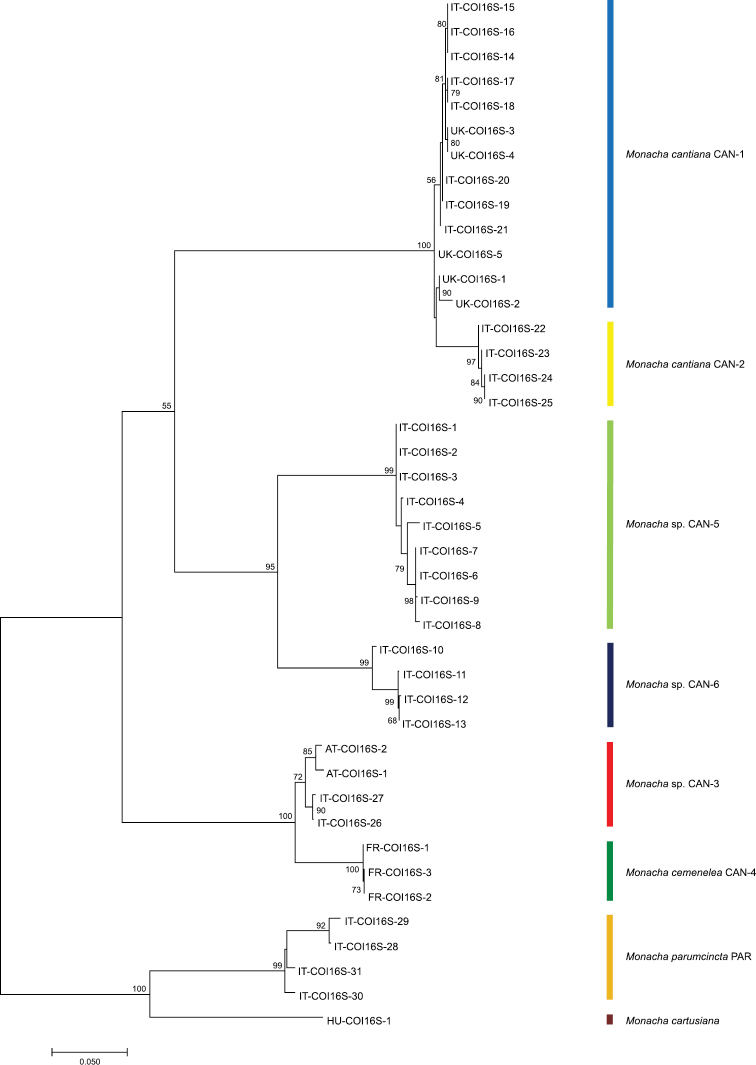
Maximum Likelihood (ML) tree of combined COI and 16SrDNA haplotypes of *Monachacantiana* s.l. (see Table 4). Numbers next to the branches indicate bootstrap support above 50% calculated for 1000 replicates (Felsenstein 1985). The tree was rooted with *M.cartusiana* and *M.parumcincta* combined sequences obtained from GenBank (Table 4).

**Figure 2.**
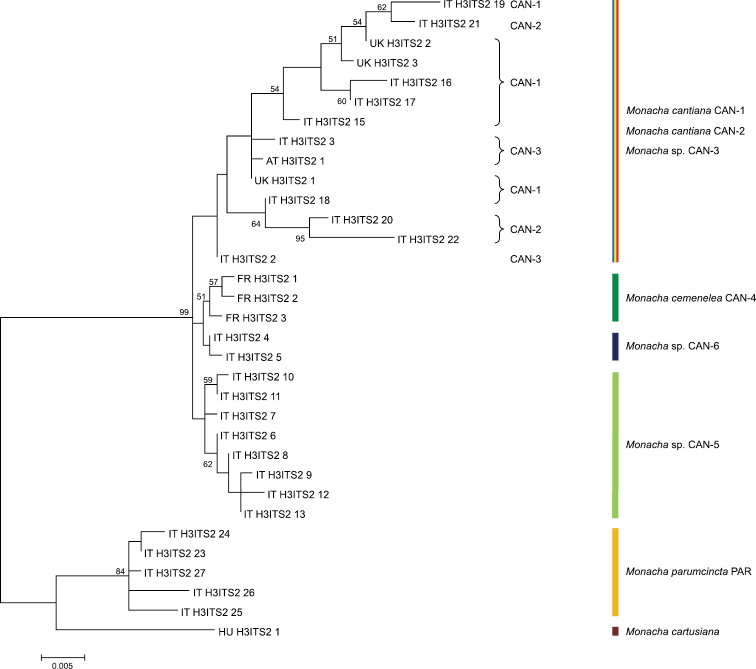
Maximum Likelihood (ML) tree of combined H3 and ITS2 common sequences of *Monachacantiana* s.l. (see Table 4). Numbers next to the branches indicate bootstrap support above 50% calculated for 1000 replicates (Felsenstein 1985). The tree was rooted with *M.cartusiana* and *M.parumcincta* combined sequences obtained from GenBank (Table 4).

**Figure 3.**
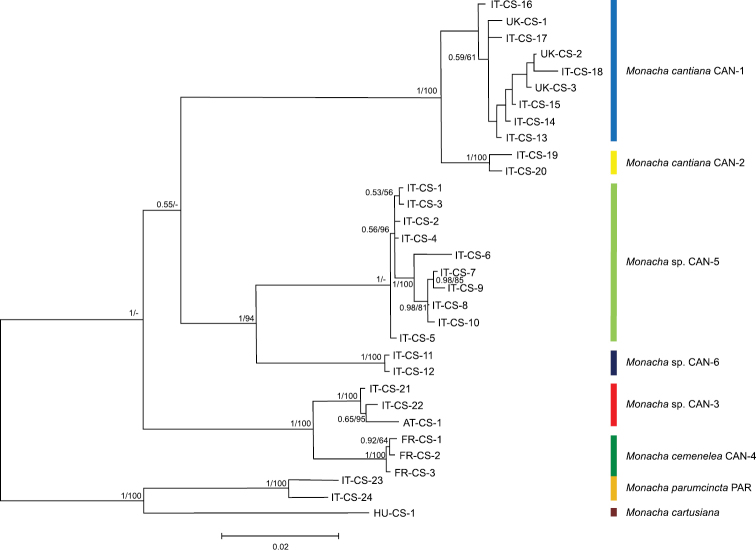
Bayesian 50% majority-rule consensus tree of the combined data set of COI and 16SrDNA haplotypes, and H3 and ITS2 common sequences (see Table 4). Posterior probabilities (left) and bootstrap support above 50% from ML analysis (right) are indicated next to the branches. Bootstrap analysis was run with 1000 replicates (Felsenstein 1985). The tree was rooted with *M.cartusiana* and *M.parumcincta* combined sequences obtained from GenBank (Table 4).

Eighteen sequences of each mitochondrial gene fragment (COI and 16SrDNA) as well as 16 and 25 sequences of nuclear gene fragments (H3 and ITS2, respectively) were deposited in GenBank as MK066929-MK066946 (COI), MK066947-MK066964 (16SrDNA), MK066965-MK066980 (H3) and MK066981-MK067005 (ITS2). Eight COI and 12 16SrDNA haplotypes were recognised among them (Table 1). Eight H3 (Table 1) and 19 ITS2 (Tables 1, 2) common nucleotide sequences were also established. ML trees for combined sequences of mitochondrial COI and 16SrDNA (Fig. 1, Table 4) and of nuclear H3 and ITS2 (Fig. 2, Table 4) gene fragments, as well as the Bayesian phylogenetic tree of combined sequences of COI+16SrDNA+H3+ITS2 gene fragments (Fig. 3, Table 4) clustered the combined sequences in two separate clades (CAN-5 and CAN-6), which were also separate from all other clades recognised previously for *M.cantiana* (CAN-1, CAN-2, CAN-3), *M.cemenelea* (CAN-4) and *M.parumcincta* (PAR) populations (Pieńkowska et al. 2018).

**Figure 4.**
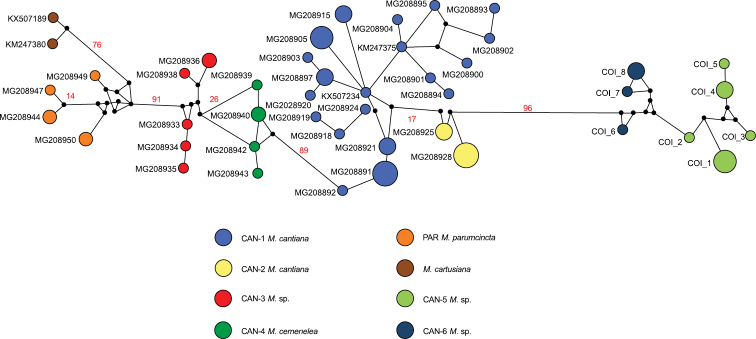
The median-joining haplotype network for COI haplotypes of *Monachacantiana* s.l. The colours of the circles indicate *Monacha* species, and their size is proportional to haplotype frequencies. Small black circles are hypothetical missing intermediates. The numbers next to the branches indicate distance between taxa expressed in numbers of mutant positions. Only numbers above 10 are indicated.

**Figure 5.**
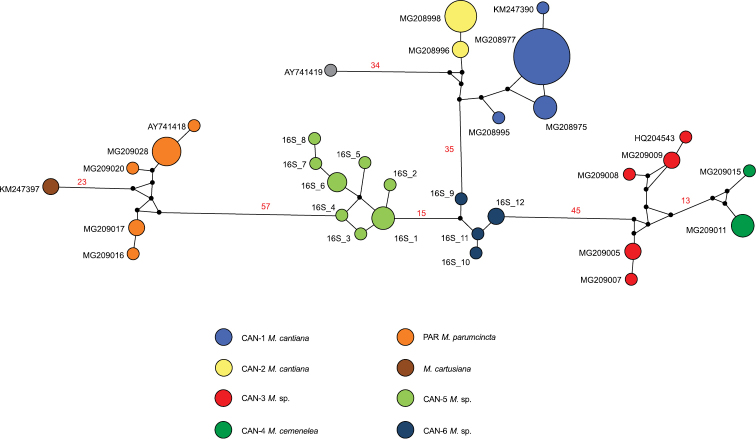
Haplotype network for 16SrDNA of *Monachacantiana* s.l. Other explanations as in Figure 4.

Networks of COI (Fig. 4) and 16SrDNA (Fig. 5) confirmed separateness of clades CAN-5 and CAN-6 and all other previously recognised clades (CAN-1 to CAN-4, PAR; Pieńkowska et al. 2018).

K2P genetic distances between COI haplotypes are summarised in Table 5. The smallest distances are within haplotypes of particular clades (0.2–2.2%, slightly larger 1.0–4.2% within *M.parumcincta*). As shown previously (Pieńkowska et al. 2018), the K2P distances between CAN-1 and CAN-2, and between CAN-3 and CAN-4, were smaller (3.3–5.1% and 5.1–6.2%, respectively) than between other clades compared in pairs (Table 5). The clades CAN-5 and CAN-6 differed considerably (12.4–14.3%). The clade CAN-5 differed to a similar degree from CAN-3 and CAN-4 clades (13.3–15.4%). Differences between these two clades (CAN-3 and CAN-4) and the clade CAN-6 were even larger (14.3–16.8%). Both CAN-5 and CAN-6 were also separated by very large genetic distances from all other clades (16.5–21.3%).

**Table 5.** Ranges of K2P genetic distances for COI sequences analysed (mean values in parentheses).

**Comparison**	**COI (%)**
Within *M.cantiana* CAN-1	0.2–2.2 (0.8)
Within *M.cantiana* CAN-2	0.3 (0.3)
Within *M.* sp. CAN-3	0.2–1.9 (1.2)
Within *M.cemenelea* CAN-4	0.2–0.5 (0.3)
Within *M.* sp. CAN-5	0.2–1.7 (1.3)
Within *M.* sp. CAN-6	0.2–2.2 (1.6)
Within *M.parumcincta*	1.0–4.2 (3.0)
Within *M.cartusiana*	0.5
Between *M.cantiana* CAN-1 and *M.cantiana* CAN-2	3.3–5.1 (3.9)
Between *M.cantiana* CAN-1 and *M.* sp. CAN-3	17.6–19.2 (18.6)
Between *M.cantiana* CAN-1 and *M.cemenelea* CAN-4	17.2–18.7 (18.0)
Between *M.cantiana* CAN-1 and *M.* sp. CAN-5	16.5–18.2 (17.5)
Between *M.cantiana* CAN-1 and *M.* sp. CAN-6	18.0–19.2 (18.6)
Between *M.cantiana* CAN-1 and *M.parumcincta*	19.6–21.7 (20.7)
Between *M.cantiana* CAN-1 and *M.cartusiana*	18.9–20.5 (19.7)
Between *M.cantiana* CAN-2 and *M.* sp. CAN-3	17.8–18.2 (18.1)
Between *M.cantiana* CAN-2 and *M.cemenelea* CAN-4	18.2–18.7 (18.5)
Between *M.cantiana* CAN-2 and *M.* sp. CAN-5	17.6–18.2 (17.9)
Between *M.cantiana* CAN-2 and *M.* sp. CAN-6	18.3–19.0 (18.5)
Between *M.cantiana* CAN-2 and *M.parumcincta*	19.8–20.7 (20.2)
Between *M.cantiana* CAN-2 and *M.cartusiana*	21.4
Between *M.* sp. CAN-3 and *M.cemenelea* CAN-4	5.1–6.2 (5.6)
Between *M.* sp. CAN-3 and *M.* sp. CAN-5	13.3–14.4 (13.8)
Between *M.* sp. CAN-3 and *M.* sp. CAN-6	14.3–16.7 (15.7)
Between *M.* sp. CAN-3 and *M.parumcincta*	18.4–21.4 (19.6)
Between *M.* sp. CAN-3 and *M.cartusiana*	18.4–20.0 (19.1)
Between *M.cemenelea* CAN-4 and *M.* sp. CAN-5	14.8–15.4 (15.1)
Between *M.cemenelea* CAN-4 and *M.* sp. CAN-6	16.4–16.8 (16.6)
Between *M.cemenelea* CAN-4 and *M.parumcincta*	19.5–20.5 (19.9)
Between *M.cemenelea* CAN-4 and *M.cartusiana*	18.9–19.3 (19.0)
Between *M.* sp. CAN-5 and *M.* sp. CAN-6	12.4–14.3 (13.6)
Between *M.* sp. CAN-5 and *M.parumcincta*	17.3–20.2 (18.5)
Between *M.* sp. CAN-5 and *M.cartusiana*	20.6–21.3 (21.1)
Between *M.* sp. CAN-6 and *M.parumcincta*	17.6–19.1 (18.2)
Between *M.* sp. CAN-6 and *M.cartusian*a	17.3–17.8 (17.5)

### Morphological study: shell

The two new clades of *M.cantiana* s.l. (CAN-5, CAN-6: Figs 6–15) have a globose-subglobose shell, variable in size and usually whitish or pale yellowish, with slightly descending, roundish to oval aperture, very similar to those of the other lineages (CAN-1, CAN-2, CAN-3, CAN-4; see Pieńkowska et al. 2018: figs 8–15), but clearly distinguished by a larger, very open umbilicus.

**Figures 6–15.**
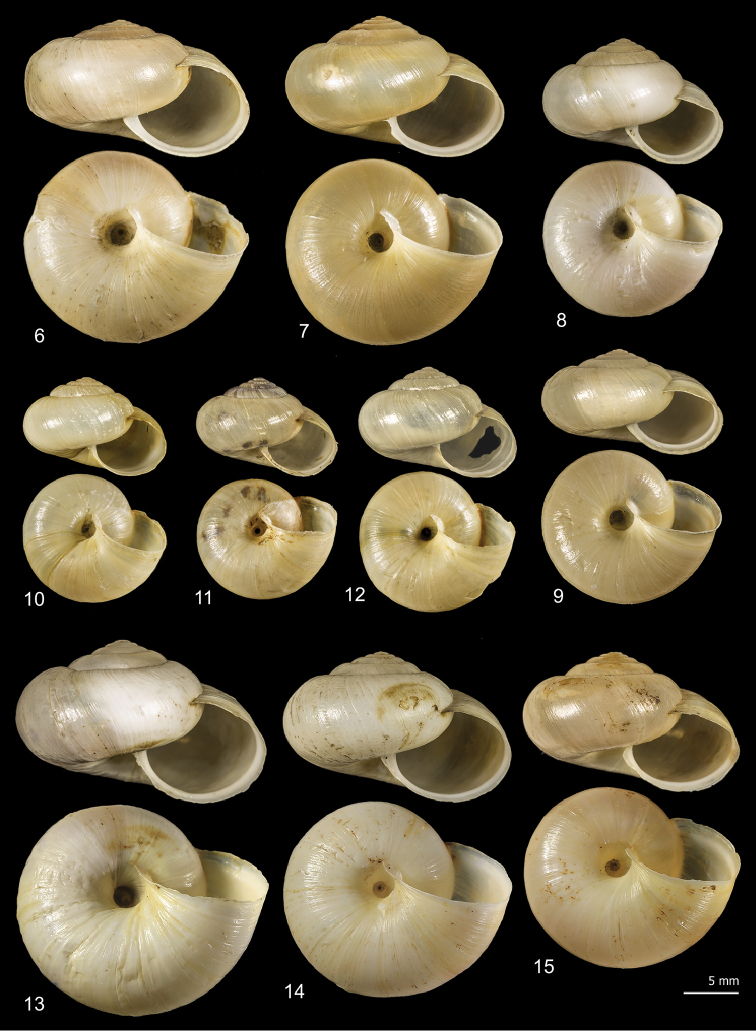
Shell variability in *Monachacantiana* s.l. CAN-5 from Piastra (FGC 41563) (**6, 7**), Foce di Pianza (FGC 41565) (**8, 9**) and Campo Cecina (FGC 41564) (**10–12**); CAN-6 from Campagrina (FGC 40322) (**13–15**).

*M.cantiana* s.l. (lineages CAN-1 to CAN-6) is always distinguished from *M.parumcincta* by its umbilicus (open in *M.cantiana* s.l.; closed in *M.parumcincta*). Some populations of *M.parumcincta* have variably evident whitish peripheral and subsutural bands (evident if the last whorl is reddish) and/or a less glossy (more opaque) shell surface.

**Figures 16, 17.**
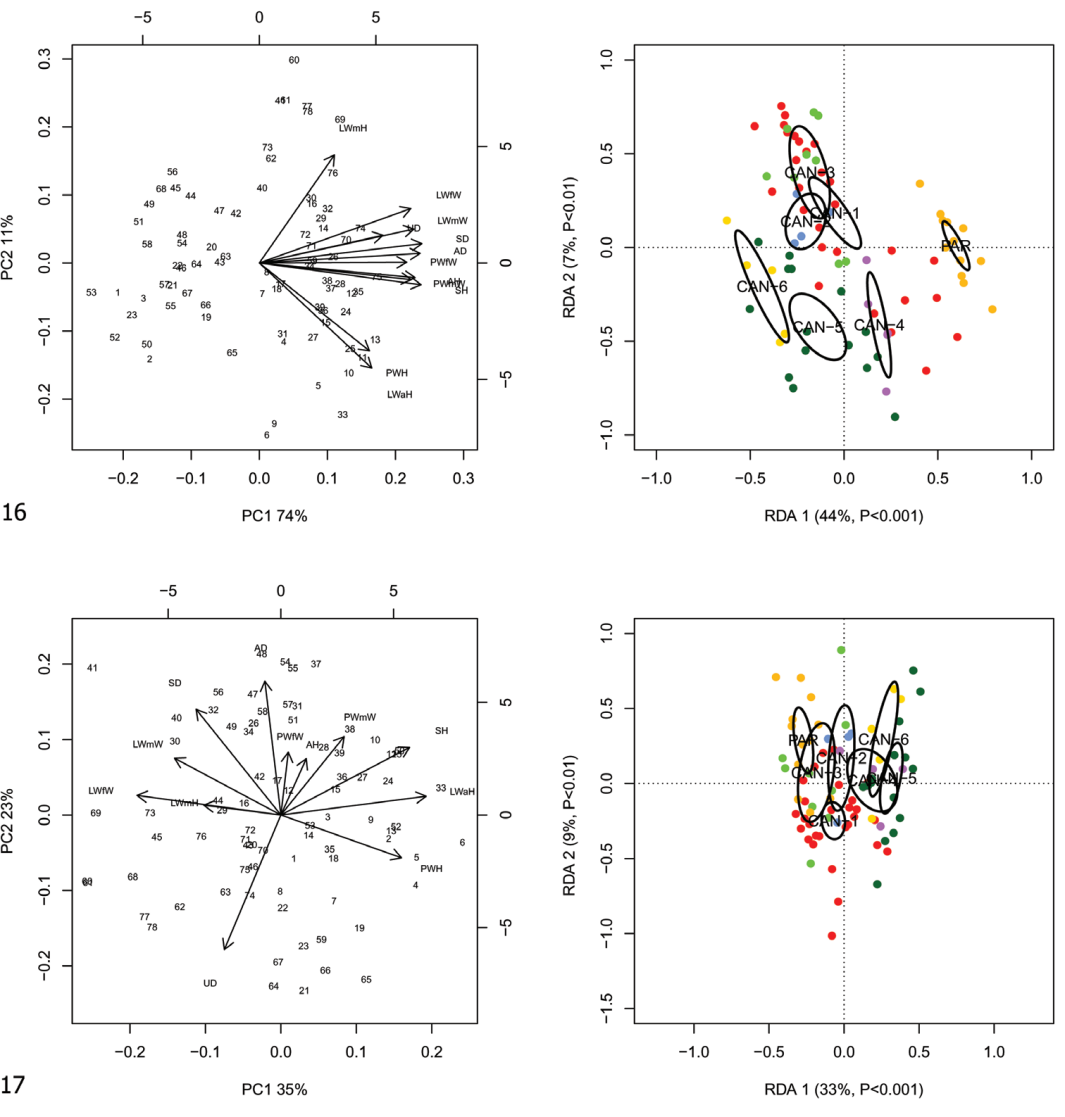
Principal component analysis (PCA) and Redundancy analysis (RDA) with lineage constraint applied to the original shell matrix (**16**) and Z-matrix (shape-related) (**17**).

RDA with lineage constraint on the shape and size matrix (Fig. 16) showed that RDA 1 (44%, *p* < 0.001) separated the groups CAN-1, CAN-2, CAN-3, CAN-4, CAN-5 and CAN-6 from PAR. The preliminary classic PCA revealed size as the first major source of morphological variation, since PC1 (74%) was a positive combination of all variables. On the contrary, RDA 2 (7%, *p* < 0.01) separated CAN-1, CAN-2 and CAN-3 from CAN-4, CAN-5 and CAN-6 with PAR in intermediate position. In this regard, PC2 (11%) accounted for a contrast between LWmH vs LWaH and PWH variables.

RDA on the shape (Z) matrix (Fig. 17) showed no separation of lineages, confirming that size is a major source of morphological variation. Shape-related PCA indicated that LWfW and LWmW vs SH, LWaH and PWH were the two principal shape determinants on PC1 and AD vs UD on PC2.

**Figure 18.**
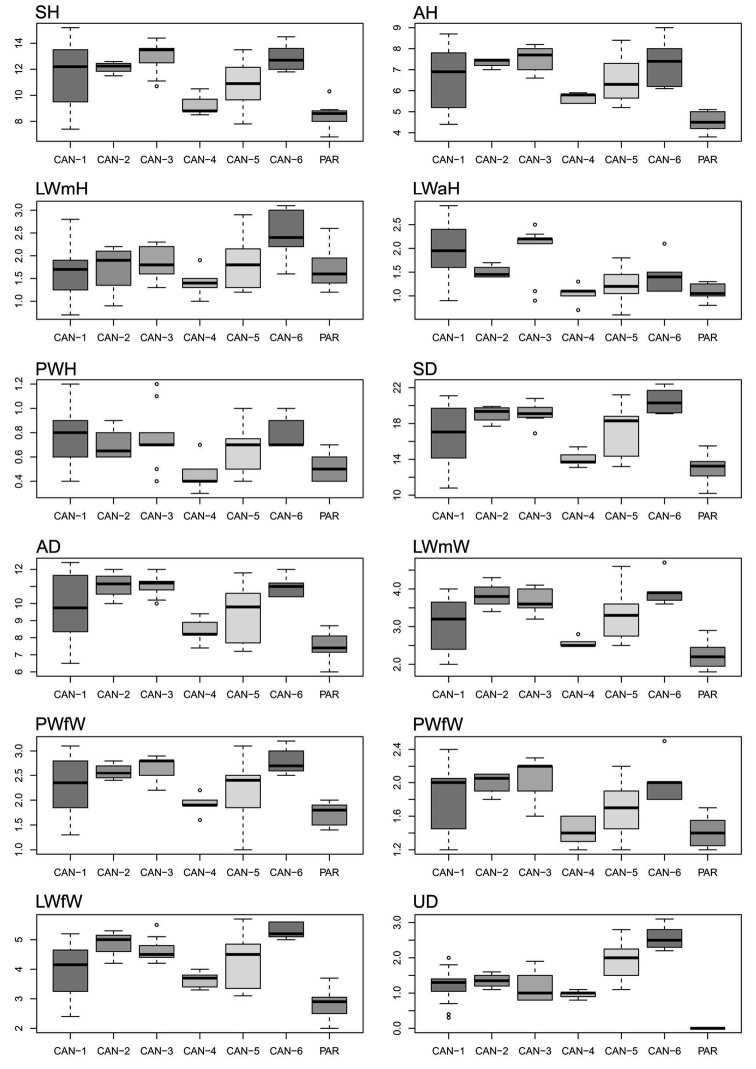
Box plots for shell characters of the seven *Monacha* clades investigated. The lower and upper limits of the rectangular boxes indicate the 25^th^ to 75^th^ percentile range, and the horizontal line within the boxes is the median (50^th^ percentile).

Box plots (Fig. 18) proved the poor discriminating value of shell characters in distinguishing lineage pairs. The best discriminant character was UD that distinguished 13 clade pairs according to Tukey’s honestly significant difference test, followed by LWmH and LWmW that distinguished seven clade pairs each. The most recognizable pairs were CAN-1 vs PAR, CAN-3 vs PAR, CAN-6 vs PAR, CAN-2 vs PAR and CAN-5 vs PAR (12, 11, 10, 8 and 7 significant characters, respectively). Five significant shell characters distinguished CAN-3 vs CAN-4, four CAN-4 vs CAN-6, two CAN-1 vs CAN-4, CAN-1 vs CAN-5 or CAN-3 vs CAN-5 and only one CAN-1 vs CAN-6, CAN-2 vs CAN-6, CAN-3 vs CAN-6, CAN-4 vs CAN-5 or CAN-4 vs PAR. No significant character distinguished CAN-1 vs CAN-2, CAN-1 vs CAN-3, CAN-2 vs CAN-3, CAN-2 vs CAN-4, CAN-2 vs CAN-5 or CAN-5 vs CAN-6 (Table 6).

**Table 6.** Results of Tukey’s honestly significant difference (HSD) test for shell and genitalia characters (in bold Tukey’s post-hoc p ≤ 0.01).

**Pairs**	**SH**	**AH**	**LWmH**	**LWaH**	**PWH**	**SD**
CAN-1 vs CAN-2	0.99624	0.80619	0.13492	0.64537	0.99057	0.63122
CAN-1 vs CAN-3	0.52140	0.28168	0.06284	1.00000	0.99999	0.22745
CAN-1 vs CAN-4	0.08096	0.59307	0.54497	**0.00097**	**0.00582**	0.34307
CAN-1 vs CAN-5	0.81752	0.99959	0.86439	**0.00006**	0.44707	0.99988
CAN-1 vs CAN-6	0.77627	0.80465	0.02347	0.29268	0.99992	0.08726
CAN-1 vs PAR	**0.00001**	**0.00000**	**0.00009**	**0.00001**	**0.00125**	**0.00032**
CAN-2 vs CAN-3	0.99544	0.99999	0.99929	0.77166	0.99881	1.00000
CAN-2 vs CAN-4	0.15929	0.22915	0.01822	0.55297	0.33334	0.07297
CAN-2 vs CAN-5	0.82169	0.71176	0.57227	0.84890	0.99950	0.79654
CAN-2 vs CAN-6	0.99776	1.00000	0.99993	0.99994	0.98407	0.99242
CAN-2 vs PAR	**0.00365**	**0.00008**	**0.00002**	0.51420	0.51214	**0.00095**
CAN-3 vs CAN-4	**0.00643**	0.04670	**0.01004**	**0.00675**	0.03910	0.01412
CAN-3 vs CAN-5	0.10929	0.23103	0.60853	**0.00526**	0.85885	0.48747
CAN-3 vs CAN-6	1.00000	0.99988	0.97726	0.47647	0.99950	0.98207
CAN-3 vs PAR	**0.00000**	**0.00000**	**0.00000**	**0.00068**	0.04033	**0.00001**
CAN-4 vs CAN-5	0.53531	0.81117	0.17929	0.96835	0.24318	0.30059
CAN-4 vs CAN-6	0.02495	0.21289	**0.00350**	0.68422	0.03827	**0.00540**
CAN-4 vs PAR	0.94161	0.19901	0.70423	0.99998	0.99243	0.94050
CAN-5 vs CAN-6	0.30662	0.70539	0.24510	0.94331	0.73973	0.19886
CAN-5 vs PAR	**0.00429**	**0.00004**	**0.00001**	0.97460	0.33336	**0.00072**
CAN-6 vs PAR	**0.00009**	**0.00003**	**0.00000**	0.65314	0.05065	**0.00001**
**Pairs**	**AD**	**LWmW**	**PWmW**	**PWfW**	**LWfW**	**UD**
CAN-1 vs CAN-2	0.69737	0.13492	0.93036	0.87269	0.31096	0.96096
CAN-1 vs CAN-3	0.31086	0.06284	0.60648	0.41696	0.21613	0.99999
CAN-1 vs CAN-4	0.50802	0.54497	0.09498	0.68052	0.97680	0.88793
CAN-1 vs CAN-5	0.96922	0.86439	0.80483	0.97841	0.92956	**0.00001**
CAN-1 vs CAN-6	0.64832	0.02347	0.86310	0.28589	0.01739	**0.00000**
CAN-1 vs PAR	**0.00015**	**0.00009**	**0.00253**	**0.00752**	**0.00003**	**0.00000**
CAN-2 vs CAN-3	1.00000	0.99929	1.00000	1.00000	0.99951	0.95368
CAN-2 vs CAN-4	0.13909	0.01822	0.07501	0.33305	0.22490	0.65706
CAN-2 vs CAN-5	0.41336	0.57227	0.53801	0.63842	0.76317	0.27349
CAN-2 vs CAN-6	1.00000	0.99993	1.00000	0.99559	0.99073	**0.00493**
CAN-2 vs PAR	**0.00086**	**0.00002**	0.01749	0.02031	**0.00004**	**0.00000**
CAN-3 vs CAN-4	0.03838	**0.01004**	**0.01014**	0.09468	0.21544	0.97116
CAN-3 vs CAN-5	0.11621	0.60853	0.13645	0.18479	0.82554	**0.00061**
CAN-3 vs CAN-6	1.00000	0.97726	1.00000	0.99741	0.83628	**0.00001**
CAN-3 vs PAR	**0.00001**	**0.00000**	**0.00029**	**0.00030**	**0.00000**	**0.00000**
CAN-4 vs CAN-5	0.89567	0.17929	0.58669	0.95667	0.75219	**0.00034**
CAN-4 vs CAN-6	0.11242	**0.00350**	0.04140	0.06153	0.02534	**0.00000**
CAN-4 vs PAR	0.78586	0.70423	1.00000	0.96612	0.13925	**0.00000**
CAN-5 vs CAN-6	0.35200	0.24510	0.38979	0.13051	0.16182	0.17535
CAN-5 vs PAR	0.01180	**0.00001**	0.19674	0.14546	**0.00001**	**0.00000**
CAN-6 vs PAR	**0.00030**	**0.00000**	**0.00588**	**0.00062**	**0.00000**	**0.00000**
**Pairs**	**DBC**	**V**	**F**	**E**	**P**	**VA**
CAN-1 vs CAN-2	0.07018	0.99978	0.78435	0.11949	0.17040	**0.00083**
CAN-1 vs CAN-3	0.95915	0.99932	0.98006	0.74183	0.08763	0.23114
CAN-1 vs CAN-4	0.99996	0.63222	0.22100	0.81959	0.76747	0.89555
CAN-1 vs CAN-5	0.94079	0.99983	**0.00000**	0.23792	0.98466	0.98588
CAN-1 vs CAN-6	0.21936	0.02524	**0.00000**	0.84359	1.00000	0.13261
CAN-1 vs PAR	0.95468	**0.00603**	0.01845	**0.00032**	0.98841	**0.00000**
CAN-2 vs CAN-3	0.59703	0.99388	0.99743	0.91922	1.00000	0.48744
CAN-2 vs CAN-4	0.22526	0.62669	0.04688	0.04004	0.04443	0.29982
CAN-2 vs CAN-5	0.01390	0.99642	**0.00000**	0.98147	0.55615	**0.00027**
CAN-2 vs CAN-6	1.00000	0.04898	**0.00000**	0.97601	0.52105	0.95169
CAN-2 vs PAR	0.02181	0.16528	0.84806	**0.00000**	0.08682	**0.00000**
CAN-3 vs CAN-4	0.96675	0.90393	0.11396	0.27618	0.02653	0.99623
CAN-3 vs CAN-5	0.60068	1.00000	**0.00000**	0.99937	0.42618	0.08653
CAN-3 vs CAN-6	0.78328	0.14420	**0.00000**	1.00000	0.43860	0.99411
CAN-3 vs PAR	0.64853	0.01508	0.39875	**0.00006**	0.04538	**0.00000**
CAN-4 vs CAN-5	0.99962	0.81255	**0.00036**	0.08838	0.48386	0.65711
CAN-4 vs CAN-6	0.37610	0.86820	**0.00508**	0.37200	0.91204	0.91815
CAN-4 vs PAR	0.99956	**0.00208**	**0.00054**	0.48361	0.98179	**0.00000**
CAN-5 vs CAN-6	0.06177	0.06806	1.00000	0.99998	0.99871	0.05266
CAN-5 vs PAR	1.00000	**0.00588**	**0.00000**	**0.00000**	0.82000	**0.00000**
CAN-6 vs PAR	0.07869	**0.00001**	**0.00000**	**0.00088**	0.99850	**0.00000**

### Morphological study: anatomy

**Figures 19–24.**
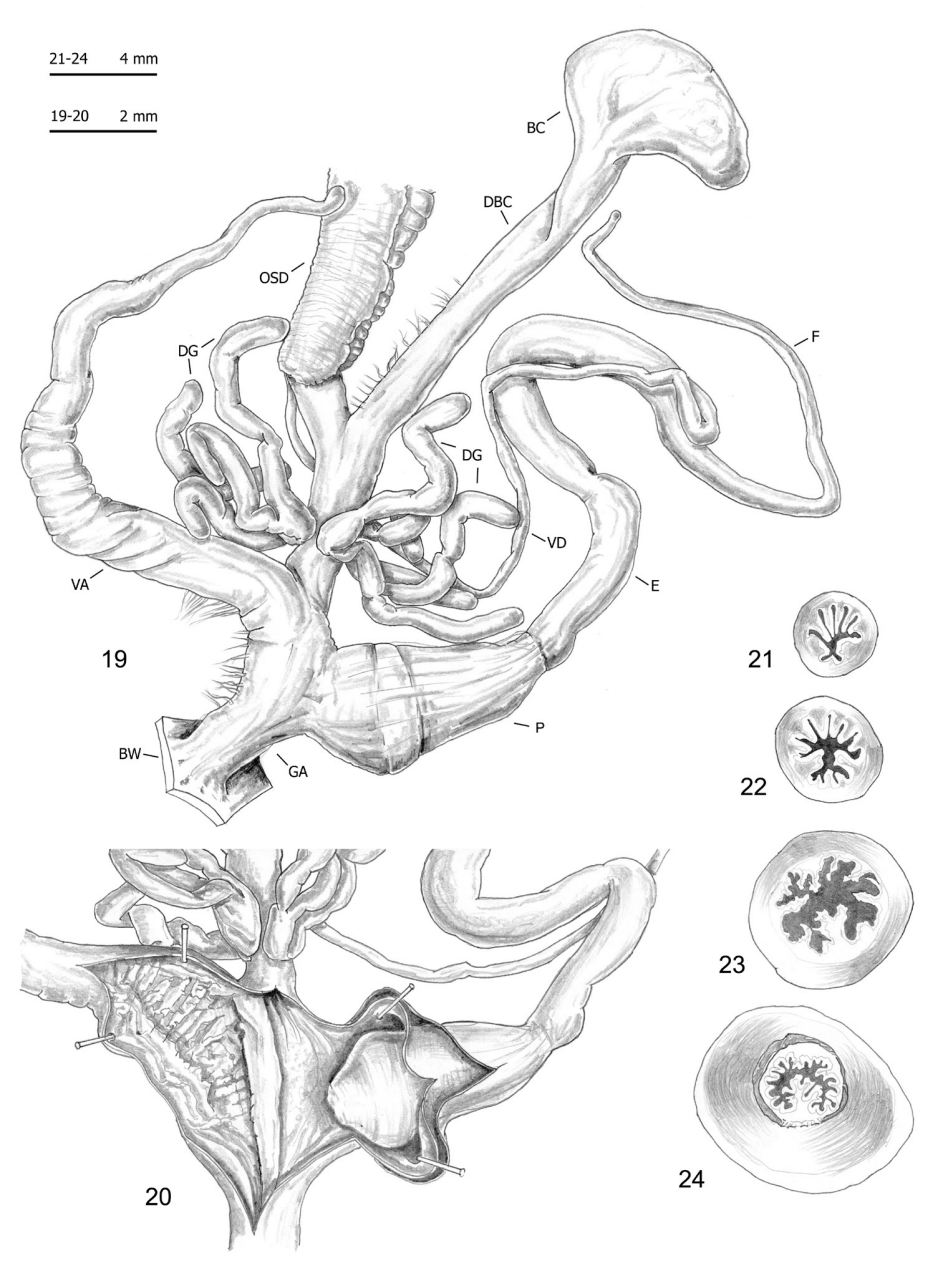
Genitalia (proximal parts excluded) (**19**), internal structure of distal genitalia (**20**), transverse sections of medial epiphallus (**21, 22**) and basal and apical penial papilla (**23, 24**) of *Monachacantiana* s.l. CAN-5 from Piastra (FGC 41563).

**Figures 25–29.**
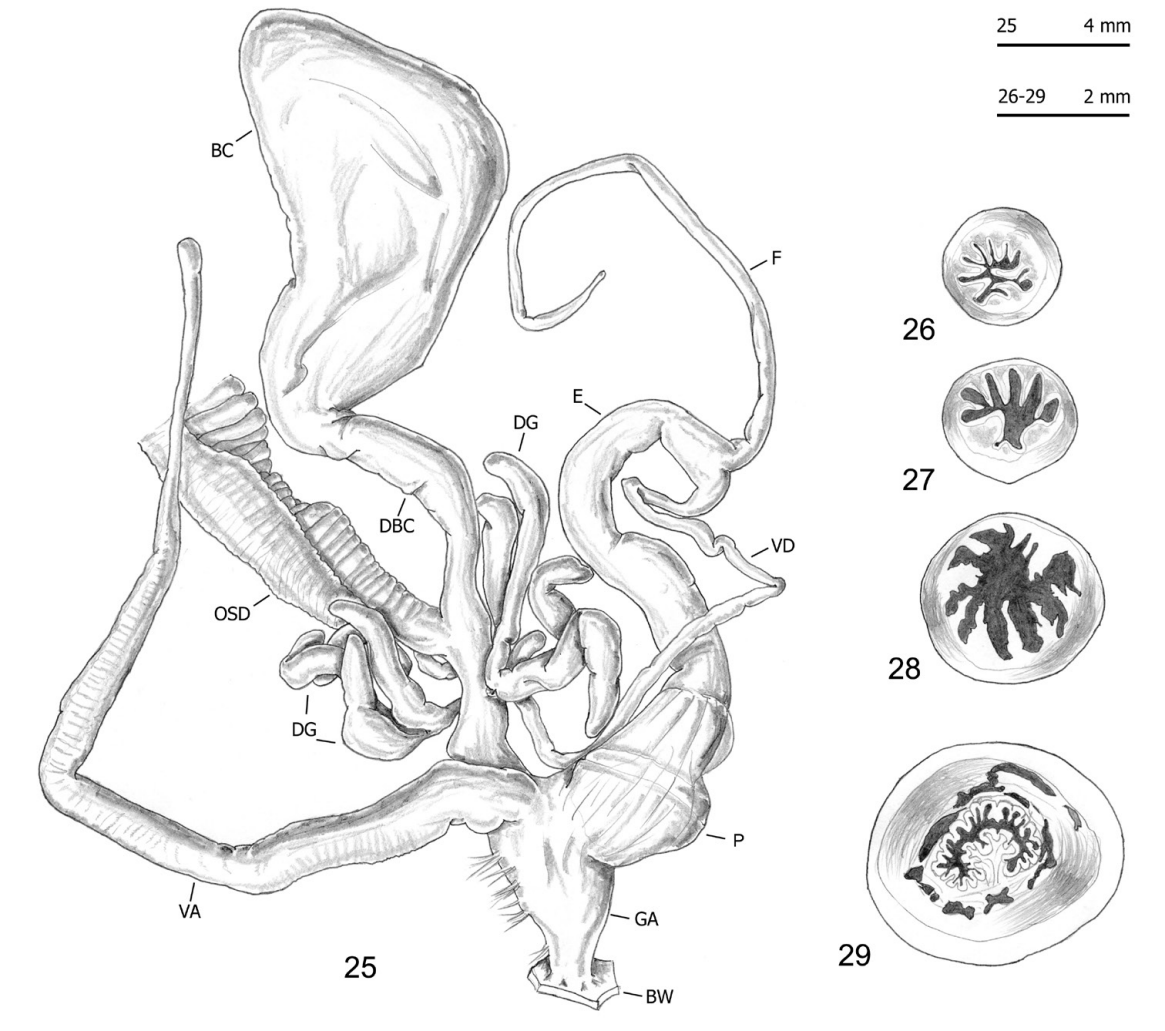
Genitalia (proximal parts excluded) (**25**), internal structure of distal genitalia (**26**), transverse sections of medial epiphallus (**27**) and basal and apical penial papilla (**28, 29**) of *Monachacantiana* s.l. CAN-5 from Foce di Pianza (FGC 41565).

**Figures 30–34.**
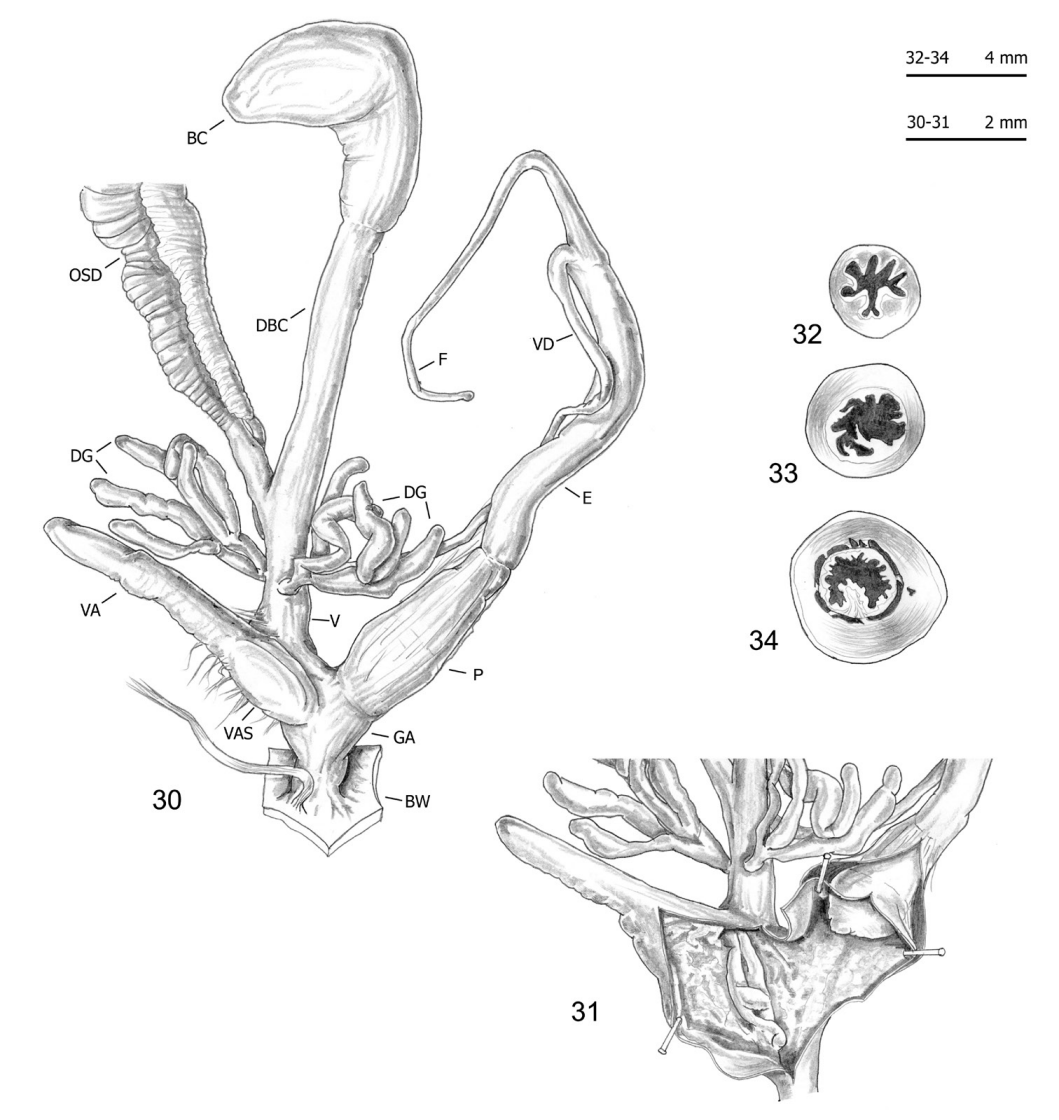
Genitalia (proximal parts excluded) (**30**), transverse sections of medial epiphallus (**31, 32**) and basal and apical penial papilla (**33, 34**) of *Monachacantiana* s.l. CAN-5 Campo Cecina (FGC 41564).

**Figures 35–41.**
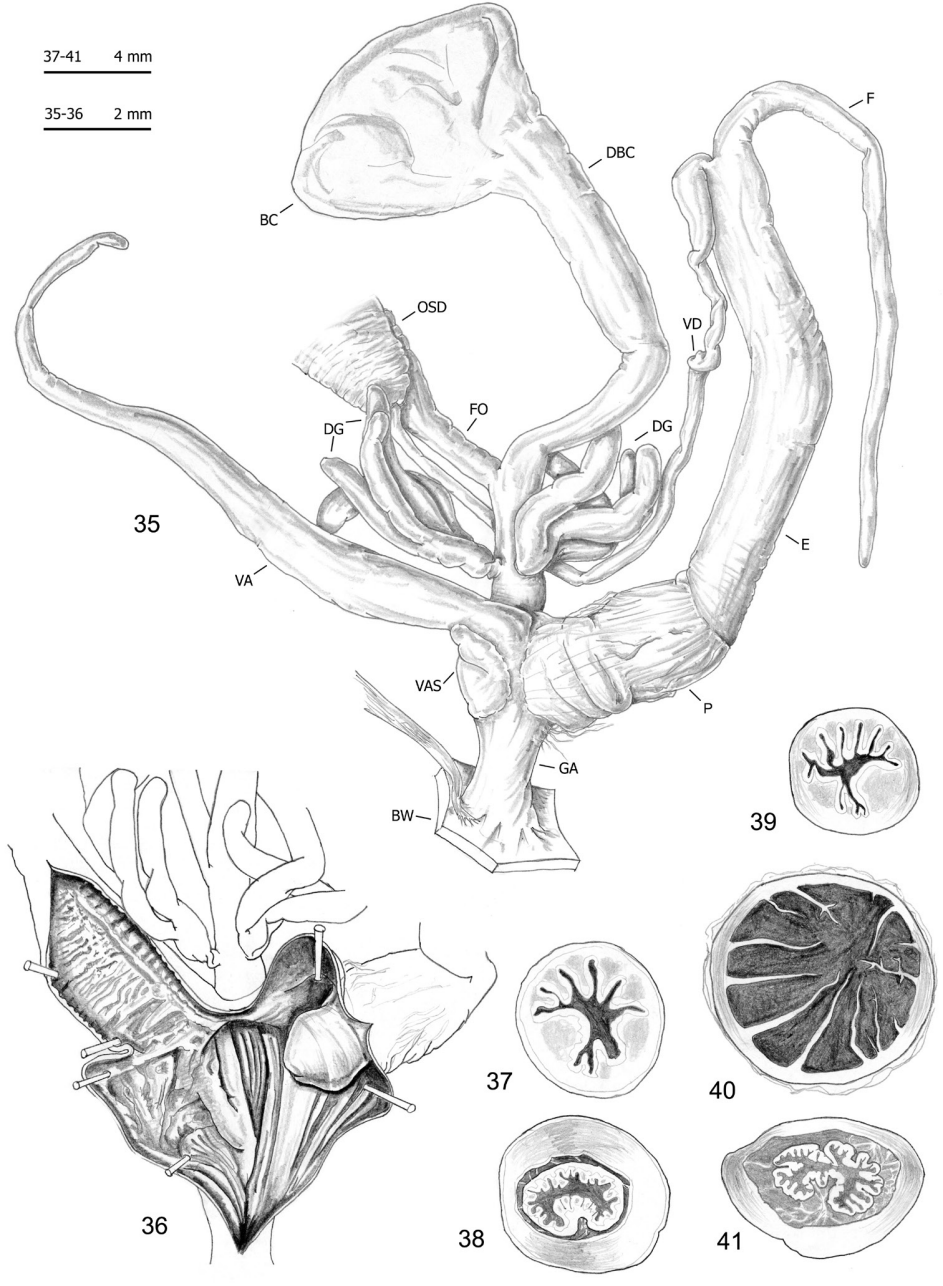
Genitalia (proximal parts excluded) (**35**), internal structure of distal genitalia (**36**) and transverse sections of medial epiphallus (**37, 39**), basal and apical penial papilla (**38, 40, 41**) of *Monachacantiana* s.l. CAN-6 from Campagrina (FGC 40322).

The bodies (generally pinkish or yellowish white) and mantle (with sparse brown or blackish spots near the mantle border or on the lung surface, a larger one close to the pneumostomal opening) of CAN-5 and CAN-6 are very similar to those of the other lineages of *M.cantiana* s.l. and *M.parumcincta* studied so far (Pieńkowska et al. 2018). The same is true of the distal genitalia (CAN-5: Figs 19–34; CAN-6: Figs 35–41), which as in the other lineages, have vaginal appendix (or “appendicula”) rather long, always with thin walled terminal portion and with variably evident basal sac; vaginal-atrial pilaster variably evident; epiphallus section with five to six small pleats on one side, two large pleats on the opposite side and, between them, a very small pleat; penial papilla (or glans) section with central canal wide, thin walled, internally irregularly jagged and with a sort of solid pilaster on one side; central canal connected to external wall of penial papilla by many muscular/connective strings as in the other lineages (Pienkowska et al. 2018).

*M.cantiana* s.l. (lineages CAN-1 to CAN-6) is always distinguished from *M.parumcincta* by its vaginal appendix (rather long with thin-walled terminal portion and variably evident basal sac in *M.cantiana*; short, only occasionally with very short terminal portion and always without basal sac in *M.parumcincta*); vaginal-atrial pilaster (present and variably evident in *M.cantiana* s.l.; absent in *M.parumcincta*); penial papilla (central canal connected to external wall by many muscular/connective strings, internally jagged and with a sort of solid pilaster on one side in *M.cantiana* s.l.; central canal not connected to external wall, internally smooth or slightly jagged and almost completely filled by large invagination in *M.parumcincta*).

**Figures 42, 43.**
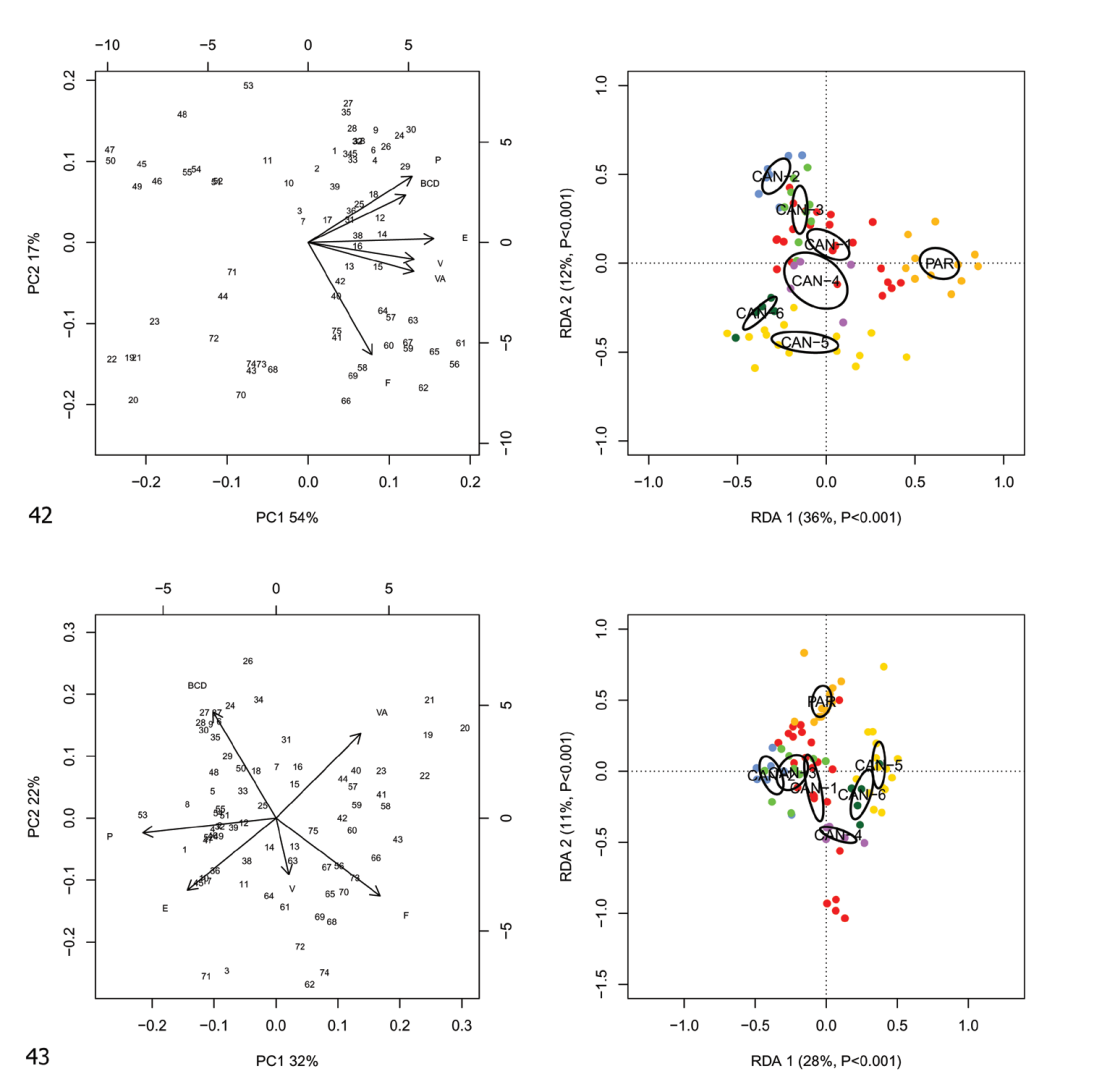
Principal component analysis (PCA) and Redundancy analysis (RDA) with lineage applied to the original genitalia matrix (**42**) and Z-matrix (shape-related) (**43**).

RDA with lineage constraint on the shape and size matrix (Fig. 42) showed that RDA 1 (36%, *p* < 0.001) separated the *M.cantiana* s.l. (CAN-1, CAN-2, CAN-3, CAN-4, CAN-5 and CAN-6) from PAR. The preliminary classic PCA revealed size as the first major source of morphological variation, since PC1 (54%) was a positive combination of all variables. On the contrary, RDA 2 (12%, *p* < 0.001) separated the group CAN-1, CAN-2, CAN-3, CAN-4 and PAR from the group CAN-5 and CAN-6. In that regard, PC2 (17%) accounted for a contrast between P and DBC vs F.

RDA with species constraint on the shape (Z) matrix (Fig. 43) showed that RDA 1 (28%, *p* < 0.001) separated the group CAN-1, CAN-2 and CAN-3 from the group CAN-5 and CAN-6 with PAR and CAN-4 in intermediate position and that RDA 2 (11%, *p* < 0.001) separated PAR from CAN-4 with the large group CAN-1, CAN-2, CAN-3, CAN-5 and CAN-6 in intermediate position. Shape-related PCA indicated that P, E and DBC vs VA and F were the two principal shape determinants on PC1 and DBC and VA vs E, V and F on PC2. In the latter case, removing the size effect altered the overall relationship patterns.

**Figure 44.**
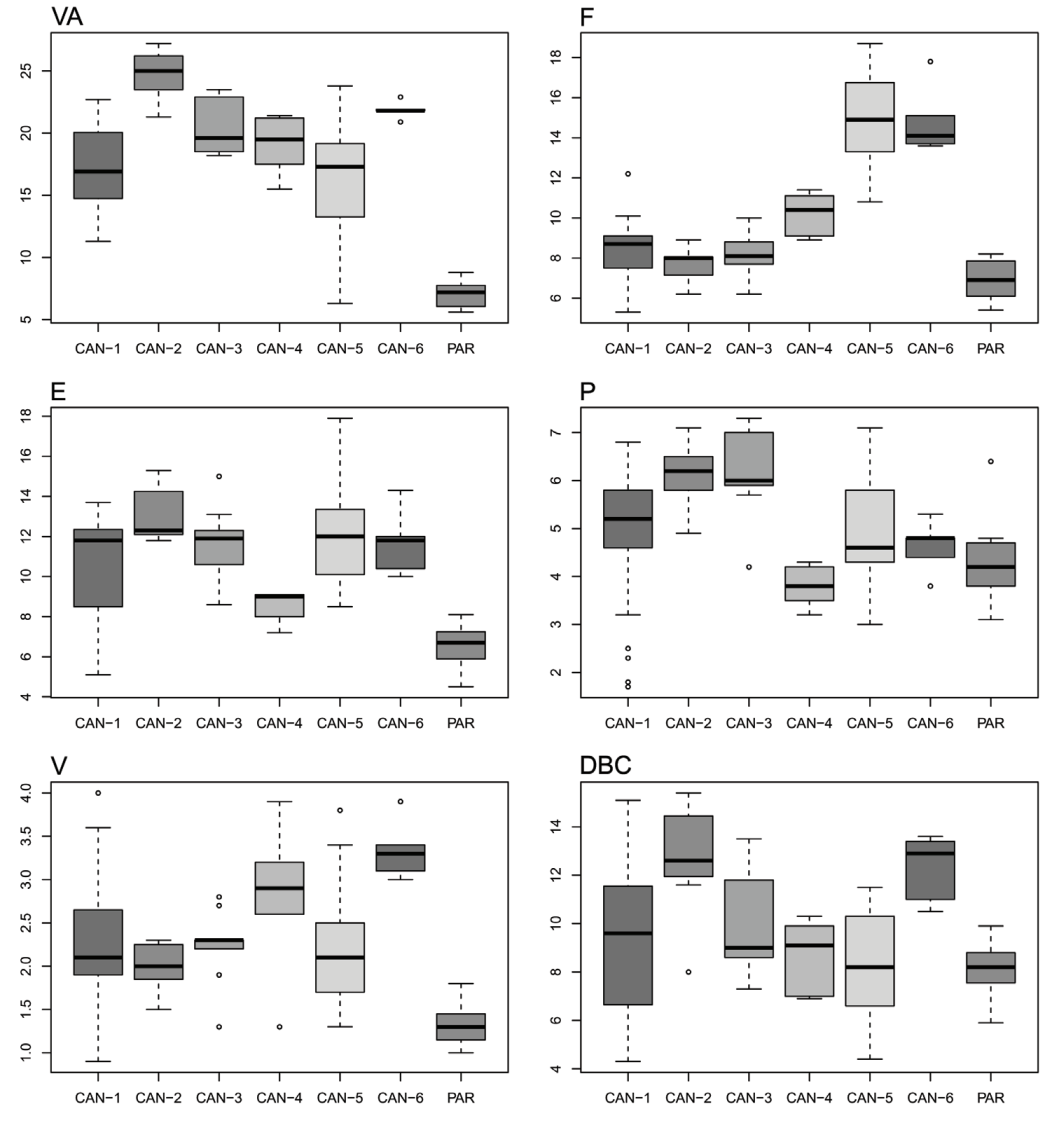
Box plots for genitalia characters of the seven *Monacha* clades investigated. The lower and upper limits of the rectangular boxes indicate the 25^th^ to 75^th^ percentile range, and the horizontal line within the boxes is the median (50^th^ percentile).

Box plots (Fig. 44) for anatomical characters showed that F and VA have the best discriminating value (they distinguished 11 and 8 clade pairs, respectively, according to Tukey’s honestly significant difference test), followed by E and V (five and four pairs, respectively). The most recognizable pairs were CAN-5 vs PAR or CAN-6 vs PAR (four significant characters), CAN-1 vs PAR or CAN-4 vs PAR (3 significant characters) and CAN-2 vs CAN-5, CAN-2 vs PAR or CAN-3 vs PAR (2 significant characters). Only one significant character distinguished CAN-1 vs CAN-2, CAN-1 vs CAN-5, CAN-1 vs CAN-6, CAN-2 vs CAN-6, CAN-3 vs CAN-5, CAN-3 vs CAN-6, CAN-4 vs CAN-5 or CAN-4 vs CAN-6 and none distinguished CAN-1 vs CAN-3, CAN-1 vs CAN-4, CAN-2 vs CAN-3, CAN-2 vs CAN-4, CAN-3 vs CAN-4 or CAN-5 vs CAN-6 (Table 6).

## Discussion

Pieńkowska et al. (2018) found that *M.cantiana*, as usually conceived, actually consists of four distinct lineages (CAN-1, CAN-2, CAN-3 and CAN-4). Examination of a group of four additional populations from the Apuan Alps revealed two more lineages (CAN-5 and CAN-6). From a molecular point of view, they are quite distinct from each other and from all the others but from a morphological point of view they are indistinguishable from each other and only slightly distinguishable from the others.

Our present results confirm that lineages CAN-1, CAN-2 and CAN-3 can be distinguished by analysis of mitochondrial gene (COI and 16SrDNA) sequences (Figs 1, 4, 5) but not by nuclear gene (H3 and ITS2) sequences (Fig. 2). On the other hand, analysis of both nucleotide sequences (of mitochondrial and nuclear genes) showed that the CAN-4, CAN-5 and CAN-6 lineages are distinct from all the others (Figs 1–3). Moreover, these gene sequences clearly separated *M.cantiana* lineages from *M.parumcincta*.

Based on their studies of lepidopteran relationships, Hebert et al. (2003a, b) suggested that nucleotide sequences of the mitochondrial COI gene could be a universal tool for species distinction. This so called “barcode method” has since been widely used (Tautz et al. 2003; Hebert et al. 2004, 2013; Hajibabaei et al. 2007; Packer et al. 2009; Goldstein and Desalle 2011; Čandek and Kuntner 2015; Dabert et al. 2018; Yang et al. 2018, but see e.g.: Moritz and Cicero 2004; Taylor and Harris 2012). It has also been used to solve taxonomic problems in different gastropod families (Hershler et al. 2003; Remigio and Hebert 2003; Rundell et al. 2004; Elejalde et al. 2008; Duda et al. 2011; Delicado et al. 2012; Breugelmans et al. 2013; Proćków et al. 2013, 2014). However, a 3% threshold was established arbitrarily by Hebert et al. (2003a, b) as a marker of species distinction, and in several stylommatophoran families it proves to be much higher (Davison et al. 2009; Sauer and Hausdorf 2010, 2012; Scheel and Hausdorf 2012). Moreover, we have always stressed (Pieńkowska et al. 2015, 2018) that molecular features alone are insufficient to define species but need to be supported by anatomical features.

In light of the above, we underline that the interspecific genetic distances in COI sequences between both, CAN-5 and CAN-6, and all other lineages of *M.cantiana* s.l. (CAN-5 vs CAN-1/CAN-2/CAN-3/CAN-4 – 13.3–18.2%, CAN-6 vs CAN-1/CAN-2/CAN-3/CAN-4 – 14.3–19.2%; Table 5) are an order of magnitude greater than Hebert’s 3% threshold (Hebert et al. 2003a, b). It is also an order of magnitude greater than intraspecific divergence (“barcode gap”, see Hebert et al. 2004; Čandek and Kuntner 2015) within CAN-5 and CAN-6 lineages, 1.3% and 1.6%, respectively (Table 5). The analysis of mitochondrial COI and 16SrDNA sequences (Figs 1, 4, 5) are supported by the results of nuclear ITS2 and H3 sequences (Fig. 2). This suggests that CAN-5 and CAN-6 lineages taken together create a taxon separate from the other lineages of *M.cantiana* s.l. Despite CAN-5 differs from CAN-6 at a similarly high level (COI 12.4–14.3%) there are no morphological differences between specimens of both lineages. The speciation of CAN-5 and CAN-6 lineages therefore seems to emerge more promptly in molecular (mitochondrial gene sequences) than in morphological (shell, genitalia) features, probably because of a rapidly evolving mitochondrial genome (Thomaz et al. 1996; Remigio and Hebert 2003). As mentioned above, molecular data alone cannot be used to distinguish species. It must be supported by morphological features of shells and/or genital anatomy before any decision is made about taxonomy or nomenclature.

Statistical analysis of 12 shell and six anatomical characters showed that CAN-5 and CAN-6 cannot be distinguished from each other by morphology (no character shows statistically significant differences according to Tukey’s honestly significant difference test). They are only marginally distinct from CAN-1, CAN-2, CAN-3 and CAN-4, but clearly distinct from *M.parumcincta*, used for comparison: two or three characters distinguish the group CAN-5 plus CAN-6 from CAN-1, CAN-2 and CAN-3; one character distinguishes CAN-5 from CAN-4; five characters distinguish CAN-6 from CAN-4; 11–14 characters distinguish the group CAN-5 plus CAN-6 from PAR. It is possible that the small sample available for lineages CAN-4 and CAN-6 (one population for each) biased comparison of these two lineages. The best discriminant characters separating the group CAN-5 plus CAN-6 from all the other lineages are umbilicus diameter (UD) and flagellum length (F). In both cases the lineages CAN-5 and CAN-6 have the highest values (Table 7).

**Table 7.** The best discriminant morphological characters distinguishing Monachacantiana lineages (UD umbilicus diameter, F flagellum length).

		**CAN-1**	**CAN-2**	**CAN-3**	**CAN-4**	**CAN-5**	**CAN-6**	**PAR**
**UD**	mean ± S.D.	1.2 ± 0.4	1.3 ± 0.2	1.2 ± 0.4	1.0 ± 0.1	1.9 ± 0.5	2.6 ± 0.4	0.0 ± 0.0
	Range	0.3–2.0	1.1–1.6	0.8–1.9	0.8–1.1	1.1–2.8	2.2–3.1	0.0–0.0
	number of specimens	28	4	9	5	15	5	12
**F**	mean ± S.D.	8.5 ± 1.5	7.6 ± 1.0	8.0 ± 1.2	10.2 ± 1.1	14.9 ± 2.5	14.9 ± 1.7	6.9 ± 1.0
	range	5.3–12.2	6.2–8.9	6.2–10.0	8.9–11.4	10.8–18.7	13.6–17.8	5.4–8.2
	number of specimens	23	7	9	5	15	5	11

All dimensions in mm.

As in the case of other lineages, the greatest bias of morphological analysis was the small sample available for lineages CAN-2, CAN-3, CAN-4 and CAN-6, which prevented a realistic account of their variability. As far as we know, this newly recognised group only occurs in the Apuan Alps and consists of two differentiated lineages (CAN-5 and CAN-6). Although examination of additional populations is desirable, intra-Apuan differentiation is also known for other organisms such as plants (Bedini et al. 2011) and animals (Zinetti et al. 2013).

Six available names have been introduced for *Monachacantiana* s.l. from north-western Tuscany (see Appendix 1). The oldest, *Helixanconae*, was established by Issel (1872) for specimens reported from a wide area extending northward to Arenzano in Liguria and southward to island of Elba and the Maremma of Tuscany. However, all the localities quoted are in coastal and lowland Liguria and Tuscany, while the populations including the group CAN-5 plus CAN-6 are from mountain sites. This would exclude a relationship of this nominal taxon with these lineages.

**Figures 45–47.**
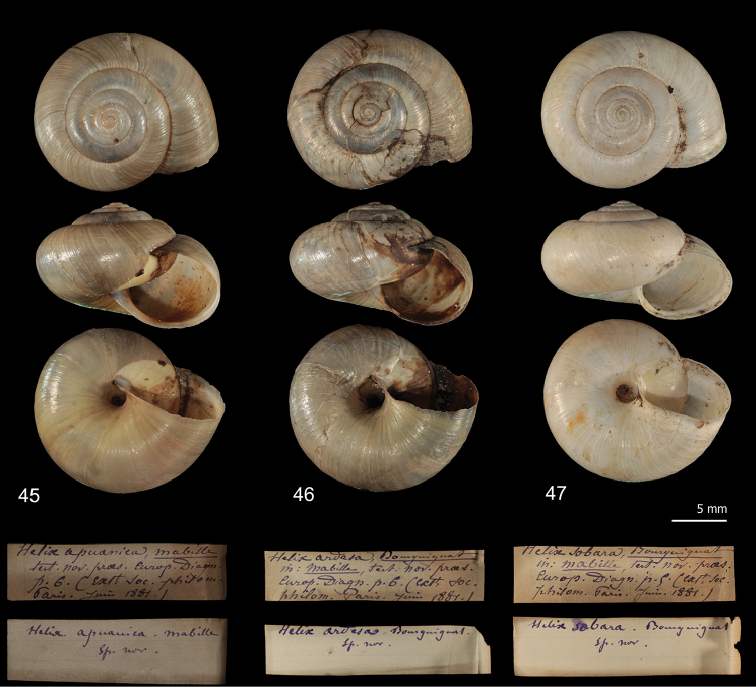
Syntypes and original labels of *Monacha* species from Apuan Alps established by Mabille (1881). *Helixapuanica* (**45**) (MHNG-MOLL-115981), *Helixardesa* (**46**) (MHNG-MOLL-115982), *Helixsobara* (**47**) (MHNG-MOLL-116022) (by courtesy of E. Tardy, Muséum d’histoire naturelle, Genève, Switzerland).

All the other names were established by Mabille (1881) and De Stefani (1883–1888) for specimens collected in the Apuan Alps. Syntypes of the three nominal taxa introduced by Mabille (1881) are in Bourguignat’s collection at the Muséum d’histoire naturelle, Genève (Switzerland) (Figs 45–47). Syntypes of the two nominal taxa established by De Stefani (1883–1888) are not known and probably lost. Umbilicus diameter of the shells of the syntypes of Mabille’s species and the specimens illustrated by De Stefani is consistent for at least five of these nominal taxa with that of *Monacha* of the group CAN-5 plus CAN-6 (*Helixsobara* Mabille, 1881, *Helixardesa* Mabille, 1881, *Helixapuanica* Mabille, 1881, *Helixcarfaniensis* De Stefani, 1883 and *Helixspallanzanii* De Stefani, 1884). Mabille’s three nominal taxa have precedence over those of De Stefani, and because the former were published simultaneously in the same paper, their relative precedence can only be determined by the first revisor (ICZN 1999: Art. 24). Although all three match *Monacha* of the group CAN-5 plus CAN-6, the best correspondence is with *Helixsobara.* Nevertheless, the availability of these names for the lineages CAN-5 and CAN-6 is somewhat difficult and not immediate. These nominal taxa were only established on shell characters, but no shell character shows statistically significant differences between CAN-5 and CAN-6. Their relationships could only therefore be established by molecular study of topotypes, but unfortunately Mabille (1881) did not quote any precise collection site. In some cases, the identity and relationships of extinct taxa have been addressed and clarified through study of ancient DNA from dried tissue (e.g. Villanea et al. 2016; Vogler et al. 2016). Unfortunately, this approach is not applicable for Mabille’s syntypes because they consist only of shells devoid of any dried tissue. Thus, the case can only be solved by appeal to article 75.5 of the Code (ICZN 1999).

However, before proposing a definitive nomenclatural taxonomic setting, it is necessary to examine other populations of the group. In the meantime, these lineages should continue to be defined informally, in order to avoid creating settings based on partial and insufficient data. This approach has also been used for other gastropods, such as *Carychiumminimum* Müller, 1774 and *Carychiumtridentatum* (Risso, 1826) (see Weigand et al. 2012), *Ancylusfluviatilis* (Müller, 1774) (see Pfenninger et al. 2003; Albrecht et al. 2006) and *Ruminadecollata* (Linnaeus, 1758) (see Prévot et al. 2016).

## Appendix 1

**Nominal taxa of *Monachacantiana* group established from north-western Tuscany**

***Helixanconae*** Issel, 1872: 63–65

Type locality: “[...] Montecatini, Val di Nievole, non lungi da Cecina nella Maremma toscana, nell’isola d’Elba, a Genova, ad Arenzano ed in altre località della Toscana e della Liguria.”

Type material: probably lost.

Status: listed as subspecies of *Monachacantiana* by Alzona (1971).

***Helixsobara*** Mabille, 1881: 126–127

Type locality: “in Alpibus Apuanis.”

Type material: one syntype (MHNG-MOLL-116022) is in Bourguignat’s collection at Muséum d’histoire naturelle, Genève (Switzerland).

Note: assigned to J. Bourguignat.

Status: listed as junior synonym of *Monachacantianaanconae* by Alzona (1971).

***Helixardesa*** Mabille, 1881: 127

Type locality: “in Alpibus Apuanis.”

Type material: one syntype (MHNG-MOLL-115982) is in Bourguignat’s collection at Muséum d’histoire naturelle, Genève (Switzerland).

Note: assigned to J. Bourguignat.

Status: listed as junior synonym of *Monachacantianaanconae* by Alzona (1971).

***Helixapuanica*** Mabille, 1881: 127–128

Type locality: “in Alpibus Apuanis.”

Type material: one syntype (MHNG-MOLL-115981) is in Bourguignat’s collection at Muséum d’histoire naturelle, Genève (Switzerland).

Note: assigned to J. Bourguignat.

Status: listed as junior synonym of *Monachacantianaanconae* by Alzona (1971).

**Helix (Monacha) carfaniensis** De Stefani, 1883: 53–54 (as “*Helixcarfaniensis*”), 1884: 231, 1888: fig. 8.

Type locality: Serchio Valley, Vagli. De Stefani (1884: 231) stated that the type is from Serchio Valley and depicted a shell from Vagli.

Type material: probably lost.

Status: listed as junior synonym of *Monachacantianaanconae* by Alzona (1971).

**Helix (Monacha) carfaniensissubvar.minor** De Stefani, 1883: 54 (as “subvar. minor”)

Type locality: “App[ennino]. San Pellegrino 1464 [m].”

Type material: probably lost.

Note: First reported by De Stefani (1875: 43–44) as Helixcantianavar.minor Albers.

Status: not available because this name denotes an infrasubspecific taxon.

**Helix (Eulota) cemeneleaformaisselii** De Stefani, 1883: 55–59 (as “Helixcemeneleaformaissellii”)

Type locality: see *Helixspallanzanii* below.

Type material: probably lost.

Status: not available because junior homonym of *Helixisseli* Morelet, 1872; renamed as *Helixspallanzanii* De Stefani, 1884.

***Helixspallanzanii*** De Stefani, 1884: 208, 231, 1888: fig. 7.

Type locality: Apuan Alps, Vagli. De Stefani (1884: 231) stated that the type is from Apuan Alps and depicted a shell from Vagli.

Type material: probably lost.

Status: new name for Helix (Eulota) cemeneleaformaisselii De Stefani, 1883, junior homonym of *Helixisseli* Morelet, 1872.

Status: listed as junior synonym of *Monachacantianaanconae* by Alzona (1971).
